# Licorice Toxicity Presenting As Refractory Hypokalemia and Hypertension in an Elderly Patient

**DOI:** 10.7759/cureus.72975

**Published:** 2024-11-04

**Authors:** Rand Albahlawan, Mohamed Alafifi, Vanessa Ambrose Fistus

**Affiliations:** 1 Internal Medicine, National Health Service, Greater Manchester, GBR; 2 Acute Medicine, Lancashire Teaching Hospitals, Lancashire, GBR

**Keywords:** electrolyte disturbances, glycyrrhetinic acid, hypertension, hypokalemia, licorice toxicity, low renin hypertension, pseudo-hyperaldosteronism

## Abstract

Licorice toxicity can present with a triad of severe hypokalemia, metabolic alkalosis, and hypertension, particularly in elderly patients. We present the intriguing case of a 78-year-old male who was referred for evaluation of refractory hypokalemia and newly developed hypertension. Despite an unremarkable systemic review and minimal symptoms, a detailed dietary history revealed significant daily consumption of licorice, initially believed by the patient to support smoking cessation. Laboratory investigations confirmed the classic biochemical profile of licorice toxicity, characterized by suppressed aldosterone and renin levels, effectively differentiating it from other conditions such as Conn's syndrome. This case underscores the diagnostic challenges posed by licorice toxicity, emphasizing the importance of a thorough clinical evaluation and awareness of dietary factors that may contribute to significant health impacts. Recognizing licorice toxicity can prevent unnecessary interventions and guide appropriate management strategies. By shedding light on this uncommon condition, we aim to enhance recognition among clinicians, avoid unnecessary interventions, and improve patient outcomes.

## Introduction

Licorice, a herb widely used for flavoring various sweets and soft drinks, is native to Asia and parts of Europe [1]. The root of licorice contains glycyrrhizin, its active ingredient, which has been linked to several cases of licorice toxicity, especially in regions where licorice is a common food staple [2]. The mechanism of licorice toxicity is thought to be pseudohyperaldosteronism, caused by glycyrrhizin's inhibition of the 11β-hydroxysteroid dehydrogenase type 2 (11β-HSD2) enzyme, which is responsible for converting active cortisol into inactive cortisone in the kidneys. This inhibition can lead to excess cortisol, which stimulates mineralocorticoid receptors and results in symptoms similar to hyperaldosteronism, such as increased sodium retention, potassium loss, and high blood pressure. This triad of hypertension, hypokalemia, and metabolic alkalosis also occurs in other conditions, such as Conn's syndrome [3]. We report a classic case of licorice toxicity, discussing its clinical features, the distinguishing factors between it and other conditions with similar presentations, and the implications for diagnosis and management.

Increasing awareness of dietary influences on health, particularly those with such significant physiological effects as glycyrrhizin, is essential for both clinicians and patients. Some studies suggest that even moderate licorice consumption can lead to toxicity, especially among individuals with predispositions to electrolyte imbalances or hypertension [4]. While licorice is often regarded as a benign herbal remedy, cases like this underscore the need for clinicians to obtain a thorough dietary history when patients present with unexpected electrolyte abnormalities. Early recognition and intervention in cases of licorice toxicity can prevent complications and reduce the likelihood of unnecessary and potentially invasive diagnostic procedures [5].

## Case presentation

Diagnosing licorice toxicity can be a challenge, demanding a detailed history that patients might not always consider relevant. This case exemplifies such complexity - a 78-year-old male was referred by his general practitioner for severe, refractory hypokalemia and recently developed hypertension. The hypertension had only been diagnosed by the patient's General Practitioner three weeks ago during a routine visit and it was being monitored after the patient started amlodipine. Despite thorough initial questioning, he reported only occasional constipation managed with osmotic and stimulant laxatives, and he denied muscle weakness, cramps, palpitations, paresthesia, or abdominal discomfort. Apart from these isolated findings, his systemic review was otherwise unremarkable, and he felt well at presentation, complicating the search for an underlying etiology.

The breakthrough came when detailed questioning revealed a seemingly unrelated but critical detail: the patient had recently begun consuming large quantities of licorice daily, believing it would aid his smoking cessation efforts. This information was initially overlooked because the patient did not consider licorice a potential contributor to his health issues, viewing it merely as a natural aid for quitting smoking. Additionally, the clinical team did not explore dietary habits thoroughly, likely due to a lack of awareness regarding licorice’s potential to induce hypokalemia and hypertension. His medical history included diverticular disease, hypertension, and dyslipidemia, and his medication regimen comprised amlodipine and atorvastatin.

Physical examination revealed a soft, non-tender abdomen with normal bowel sounds, and the cardiovascular and respiratory evaluations were unremarkable. Vital signs were stable except for an elevated blood pressure of 172/87 mmHg (Table 1).

**Table 1:** Serum potassium and blood pressure monitoring during the patient length of hospital stay. The table illustrates persistently low potassium levels despite adequate oral and IV replacement, attributed to renal losses caused by pseudohyperaldosteronism. The introduction of amiloride on day 5 led to a notable shift, resulting in improved serum potassium levels above 3 mmol/L, which allowed for a safe discharge.

Days since admission	Serum potassium level (mmol/L)	Blood pressure (mmHg)
1	2.3	172/87
2	2.5	168/98
3	2.3	166/91
4	2.4	189/91
5	2.6	168/93
6	3.0	179/92
7	3.1	176/90
8	2.8	175/88
9	2.9	163/96
10	3.0	158/85
11	3.4	135/90

Initial laboratory findings were consistent with severe hypokalemia (potassium 2.3 mmol/L), accompanied by hypernatremia (sodium 152 mmol/L) and borderline low magnesium (0.6 mmol/L). Adjusted calcium and phosphate levels were within normal limits, as were liver and renal function tests, along with infectious and inflammatory markers. Thyroid function tests showed no abnormalities. Venous blood gas analysis revealed a significant metabolic alkalosis (pH 7.54, HCO3 38.5 mmol/L). Further endocrinological workup demonstrated an elevated early morning cortisol level, with suppressed serum aldosterone levels (<145 pmol/L) and low renin activity (3.8 mIU/L), alongside elevated urinary potassium excretion (45.4 mmol/L).

Electrocardiogram (ECG) analysis revealed a normal sinus rhythm with a heart rate of 64 beats per minute, and a review of recent imaging, including a CT scan of the thorax, abdomen, and pelvis conducted one month prior, showed no significant abnormalities.

The patient received intensive intravenous potassium and magnesium supplementation. While magnesium levels were corrected promptly, potassium levels remained stubbornly low (2.3-2.6 mmol/L) despite adequate oral and intravenous therapy (Table 1). We discontinued amlodipine and introduced amiloride 10 mg once daily. Amiloride is a potassium-sparing diuretic that inhibits epithelial sodium channels (ENaC), promoting sodium excretion and reducing potassium secretion. It opposes the effects of licorice toxicity, which causes pseudohyperaldosteronism and increases sodium retention and potassium excretion through enhanced stimulation of mineralocorticoid receptors. This resulted in a notable improvement in potassium levels, which rose above 3 mmol/L, permitting a safe discharge. At follow-up, with the effects of licorice toxicity resolved, potassium levels fully normalized, and blood pressure stabilized within the target range, allowing the patient to remain off long-term antihypertensive therapy.

## Discussion

Licorice toxicity exerts its effects through a mechanism known as pseudohyperaldosteronism, primarily mediated by glycyrrhizin's inhibition of the enzyme 11-β-HSD2 [6]. Under normal circumstances, this enzyme converts cortisol into its inactive form, cortisone, thereby preventing cortisol from activating mineralocorticoid receptors. When glycyrrhizin inhibits 11-β-HSD2, cortisol levels increase, leading to unregulated activation of mineralocorticoid receptors. This activation results in sodium retention, potassium loss, and elevated blood pressure. These are classic features observed in our patient, who presented with severe hypokalemia, metabolic alkalosis, and hypertension.

A critical differentiating factor in diagnosing licorice toxicity is the suppression of both aldosterone and renin levels, a consequence of the inhibition of the renin-angiotensin-aldosterone system (RAAS). This profile was evident in our case. In contrast, conditions such as Conn's syndrome (primary hyperaldosteronism) typically present with elevated aldosterone levels and low renin due to an aldosterone-secreting adrenal adenoma [7].

In considering other conditions that mimic licorice toxicity, apparent mineralocorticoid excess (AME) arises from reduced activity of 11-ß-HSD2, albeit due to a genetic defect [8]. Like licorice toxicity, AME results in low-renin hypertension with suppressed aldosterone levels. However, the acquired nature of our patient’s condition and his older age at presentation distinguish it from AME, which is often diagnosed in younger individuals with a genetic predisposition. Genetic testing and a thorough family history can aid in differentiating between the two conditions.

Additionally, certain subtypes of congenital adrenal hyperplasia (CAH), such as 17α-hydroxylase deficiency, can present with similar biochemical findings, including low-renin hypertension and hypokalemia [9,10]. However, these genetic disorders typically involve additional clinical features such as delayed puberty, a younger age of onset, and family history, which were not noted in our patient, further steering the differential diagnosis away from CAH.

Renovascular hypertension, commonly caused by renal artery stenosis, must also be considered in cases of unexplained hypokalemia and hypertension, particularly in older patients with cardiovascular risk factors [11]. In contrast to our patient’s suppressed aldosterone and renin levels, renovascular hypertension generally exhibits elevated renin and aldosterone due to impaired renal perfusion. This coupled with the absence of significant atherosclerotic risk factors in this patient’s history also diminished the likelihood of this diagnosis. Tests that could help exclude this condition include renal arteriography, the gold standard diagnostic imaging test, which is relatively invasive [12]. However, this can be avoided by recognizing the biochemical patterns associated with licorice toxicity and obtaining the crucial details of the patient’s licorice consumption through a thorough history.

Hypercortisolism, as seen in Cushing's syndrome, results from excessive cortisol production due to adrenal tumors, pituitary adenomas, or ectopic sources, and can mimic mineralocorticoid activity, leading to sodium retention, hypokalemia, and metabolic alkalosis [8]. However, this condition typically presents with distinctive physical features such as central obesity and purple striae, which were notably absent in our patient, thereby helping to exclude this diagnosis. Additional tests, such as a 24-hour urinary-free cortisol or low-dose dexamethasone suppression test, could help exclude this condition. However, we did not consider these necessary for our patient, given his reported excessive licorice consumption and the absence of clinical signs of hypercortisolism.

Another condition to consider is Liddle syndrome, a rare genetic disorder caused by a mutation that increases the activity of ENaC in the kidneys, resulting in low-renin, low-aldosterone hypertension [13]. Diagnosis typically involves genetic testing to confirm the presence of ENaC mutations, along with a detailed family history to identify early onset or inherited patterns of hypertension [14]. These characteristics were absent in our patient, further distinguishing Liddle syndrome from the acquired condition observed.

Our patient’s significant licorice consumption strongly indicated licorice toxicity as the underlying cause of his symptoms. This diagnosis was supported by a detailed dietary history, a simple, non-invasive approach that spared the patient from numerous invasive investigations. The resolution of hypertension and potassium imbalance following the cessation of licorice intake further confirmed this diagnosis.

Reported cases of licorice toxicity are relatively uncommon but can be life-threatening. One study identified six patients who required intensive care for licorice toxicity between 2018 and 2020 in a single center [15]. All presented with arterial hypertension, hypokalemia, and metabolic alkalosis, indicating a clear pattern. Therefore, licorice toxicity should be considered when encountering this triad of symptoms. Clinicians should specifically inquire about licorice consumption in these cases, as doing so can help prevent unnecessary invasive tests and the use of long-term antihypertensive agents.

One notable case involved the ingestion of licorice root tea and presented similarly to our patient, featuring the classic triad but with a severe hypertensive emergency, illustrating the extent of licorice’s effect on blood pressure [16]. Licorice intoxication effectively results in a clinical state indistinguishable from hyperaldosteronism, which is why it is termed pseudohyperaldosteronism. However, these two conditions can be differentiated by measuring plasma renin activity and plasma aldosterone levels. Hyperaldosteronism is characterized by excessive aldosterone production leading to sodium retention, potassium loss, and hypertension, while pseudohyperaldosteronism mimics these effects through mechanisms other than elevated aldosterone, such as cortisol activating mineralocorticoid receptors.

Our patient case highlights the importance of considering licorice toxicity as a potential cause of pseudohyperaldosteronism in patients with the classic triad of hypertension, hypokalemia, and metabolic alkalosis. The resolution of symptoms upon cessation of licorice intake highlights the importance of thorough patient histories in distinguishing conditions with overlapping presentations, enabling accurate diagnosis, avoiding unnecessary interventions, and optimizing patient management.

## Conclusions

This case highlights the diagnostic challenges of identifying licorice toxicity, particularly in older patients whose dietary habits might not be immediately linked to their symptoms. The subtle onset and the patient’s initial lack of awareness regarding the effects of licorice consumption emphasize the need for a high degree of clinical suspicion. The key to diagnosing licorice toxicity lies in recognizing the characteristic suppression of both aldosterone and renin levels, a profile evident in our patient, which distinguishes it from conditions like Conn’s syndrome that present with elevated aldosterone and low renin. This suppression profile was essential in confirming licorice toxicity as the underlying cause, as it indicated cortisol's mineralocorticoid effects mimicking aldosterone activity.

Recognizing treatment-resistant hypokalemia in licorice toxicity is clinically important, as it underscores the need to consider licorice toxicity in cases of unexplained hypokalemia and hypertension, especially when standard therapies are ineffective and renin-aldosterone levels are suppressed. This case also demonstrates the effectiveness of amiloride in resolving hypokalemia, further supporting its role in management. Accurate diagnosis relies on a comprehensive approach that includes detailed dietary histories, clinical correlation, and an understanding of the biochemical profiles of potential differential diagnoses. By identifying the unique features of licorice toxicity, clinicians can implement targeted treatments that lead to significantly better patient outcomes, ultimately enhancing the quality of care for those affected.
